# Evaluation of the cost and care outcomes by group related to the diagnosis of bariatric surgery

**DOI:** 10.1186/s12893-024-02682-y

**Published:** 2024-11-29

**Authors:** Beatriz Böger, Guilherme de Souza Ribeiro, Bianca Fontana Aguiar, Jolline Lind, Anne Karine Bosetto Fiebrantz, Moacir Pires Ramos, João Henrique Felicio de Lima, Jaime Luis Lopes Rocha

**Affiliations:** 1 Research and Innovation Center, Unimed Curitiba, Av. Affonso Penna, 297, Curitiba, 82530-280 Brazil; 2 Federal University of Paraná, Av. Prefeito Lothário Meissner, 623, Curitiba, 80210-170 Brazil; 3 RG Analytcs, Caxambu Street, No. 223, Itaúna, 35.680-280 Brazil; 4 Pontifical Catholic University of Paraná, Imac. Conceição Street, Curitiba, 80215-901 Brazil; 5 Epidemiology Hub, Unimed Curitiba, Curitiba, Brazil

**Keywords:** Value-based healthcare, Diagnosis related groups, Real World data, Obesity, BMI, Bariatric surgery, Supplementary health

## Abstract

**Background:**

To conduct a comprehensive assessment of real patient data undergoing the procedure within a healthcare provider, integrating both costs and care stages related to bariatric surgery, emphasizing the relevance of analysis by Diagnosis-related group (DRG).

**Methods:**

Prospective study of patients coded by DRG within a network of providers accredited to a Brazilian healthcare provider. All patients coded with metabolic and bariatric surgery (MBS) between 01/2019 and 06/2023 and undergoing gastrectomy procedure were included for analysis. The cost base used was derived from administrative payment information of the healthcare provider. Analyses were presented as mean, median, and standard deviation. Levene, Student’s t-test, Kendall’s tau, and Pearson’s chi-square tests were used.

**Results:**

The study included a total of 1408 patients who underwent MBS in four prominent hospitals in the area during the specified period. Among these patients, an average of 74.8% were female, with a mean age of 37.31 years and a mean body mass index (BMI) of 40.3 kg/m2. Furthermore, 88.9% of the patients underwent gastric bypass. Although there were few acquired complications during hospital admission there were vascular complications following infusion, transfusion, and therapeutic injection, 22.45% (*n* = 11), hemorrhage and hematoma complicating procedure not classified elsewhere, 8.16% (*n* = 4), leakage, 8.16% (*n* = 4), and one death during this study. There were 67 readmissions within 30 days (4,75%). The total costs incurred throughout the patient’s journey, covering hospitalization and one-year post-procedure, exhibited a median value of $4,078.53. Additionally, a notable positive association was observed between post-discharge expenses and age, indicating a tendency for costs to rise as patients grow older.

**Conclusion:**

The identified results highlight the complexity and challenges associated with bariatric surgery, including patient management and substantial costs involved. Therefore, a more comprehensive and personalized approach in postoperative management and resource allocation may be necessary to optimize clinical and economic outcomes.

**Supplementary Information:**

The online version contains supplementary material available at 10.1186/s12893-024-02682-y.

## Introduction

Obesity exerts a distinct influence on overall mortality, as evidenced by the fact that the life expectancy of individuals possessing a Body Mass Index (BMI) falling within the range of 40 to 45 kg/m² experiences a reduction of approximately eight to ten years [1]. It is notable that roughly 25.6% of individuals with a BMI ≥ 40 kg/m² are afflicted by type 2 diabetes (T2D), while 50.9% suffer from arterial hypertension (AH) [1, 2]. This corresponds to a 7.4-fold increase in the risk of T2D and a 6.4-fold increase in the risk of AH compared to individuals with normal weight [2, 3]. In Western Europe, 3.3% of all cancers in men and 7.8% of all cancers in women may be related to obesity [2, 3]. In Brazil, the obese adult population is steadily increasing and reaches up to one-third of the population. The World Health Organization asserts: the “pandemic” of obesity is one of the most serious health problems we face [4, 5].

The increasing prevalence of obesity and its related complications lead to a significant increase in the volume of surgeries and the improvement of bariatric surgeries as an effective strategy for treating these conditions [2, 6–8].

Data compiled by the Brazilian Society of Bariatric and Metabolic Surgery (SBCBM) indicate that 74,738 procedures were performed in the country in 2022. The number of surgeries performed by health plans - according to a recent survey by the National Supplementary Health Agency (ANS) - was 65,256 surgeries in the same period. The growth was 22.9% compared to 2019, a year before the pandemic, when 53,087 surgeries were performed [8–10].

Patients with BMI > 40 kg/m² are potential candidates for surgical treatment, aiming at metabolic improvement and control of complications and risk factors resulting from excess weight. The metabolic and bariatric surgery (MBS) is recommended for individuals with a BMI > 35 kg/m^2^, regardless of presence, absence, or severity of co-morbidities [4, 10, 11].

In this context, analysis by Diagnosis-Related Group (DRG) emerges as a fundamental analytical tool. The DRG presents a systematic approach to classifying patients based on similar clinical and surgical characteristics, and therefore should also present similar costs. This allows for a more precise and detailed evaluation of different healthcare providers [8, 11, 12]. By applying this methodology to bariatric surgery, it is possible to identify specific costs and outcomes patterns for different patient subgroups, providing a more granular and personalized view of the care process [4, 6, 8, 13].

The complexity of this procedure, however, extends beyond purely clinical aspects, encompassing economic considerations that directly impact healthcare systems. Understanding the cost associated with bariatric surgery becomes essential to optimize resource allocation and ensure the sustainability of these interventions over time [3, 8, 13, 14].

There is a growing gap in the literature, wherein the efficacy of clinical results is increasingly understood and the frequency of the procedure rises, yet the financial implications or advantages remain ambiguous. This study aims to evaluate real patient data and costs related to bariatric surgery within a healthcare provider, emphasizing analysis by Diagnosis-Related Group (DRG).

By exploring the nuances associated with each specific patient DRG group, valuable insights are offered for healthcare managers in their strategic decision-making process, for clinical professionals in improving the quality of care, and for researchers in expanding knowledge about the outcomes of this crucial intervention. By undertaking this endeavor, our aim is to contribute to a more efficient and personalized approach in the management of bariatric surgery, benefiting both patients and healthcare systems worldwide.

## Methods

### Study design and sample

This study was a retrospective survey of patients coded by DRG in a network of healthcare providers accredited to a Brazilian health insurance company (Southern Brazil), responsible for more than 600,000 lifes. Initially, all patients coded with a primary DRG of MBS between 01/2019 and 06/2023 were included for analysis. Subsequently, those who underwent the procedure for the first time were selected for further study. There were no exclusion criteria at this point. The cost data employed in this study were obtained from administrative payment records provided by the health insurance provider. The data set(s) supporting the conclusions of this article are available in a private medical cooperative repository.

The results obtained from the study cohort were characterized by the study population, as well as information on the gastrectomy technique performed, acquired condition, length of hospital stay, readmission, death, costs involved in the perioperative period of patients, and in the one-year period after medical discharge were evaluated and treated. This study quantifies direct medical costs involved in bariatric surgeries.

### Statistical analysis

Descriptive analyses were presented as mean, median (IQR), and standard deviation (SD). Categorical variables (qualitative) were presented as frequency (percentage) (Microsoft Excel^®^ version 2016, Microsoft Corporation, USA).

Data normality was assessed by Kolmogorov-Smirnov and Shapiro-Wilk tests. The assumption of variance homogeneity was evaluated using Levene’s test. Bootstrap procedures (1000 re-samples; 95% BCa CI) were performed to obtain greater reliability of the results, to correct deviations from normality of the sample distribution and differences between group sizes [15]. To evaluate statistical differences between groups, the Student’s t-test for independent samples was used, and correlation was assessed by Kendall’s tau (τ) and Pearson’s chi-square test used to test the strength of association between two variables. Shared variance (r2) was used to assess the responsiveness of correlation. Statistical analyses were performed using SPSS software version 20 (Chicago, USA). A two-tailed significance level of *p* < 0.05 was considered statistically significant [16].

### Ethical aspects

Procedures followed standards of scientific research and were conducted in accordance with the Declaration of Helsinki [17]. Patient data were anonymously obtained from the DRG database. Therefore, this study is exempt from bioethical approval as it does not involve any intervention in human beings.

## Results

A total of 1408 patients who underwent MBS in four major hospitals in the region during the study period were included. Of these, on average, 74.8% were female, with female predominance observed in all years (Table S1). On average, 281.6 bariatric surgeries were performed per year (min. 87 and max. 471 surgeries/year).

Only 50,03% of all patients had obesity grade III or higher. Considering all included patients, 12.2% (*n* = 172) had arterial hypertension, 7.5% (*n* = 106) had long-term use of anticoagulants, 3.7% (*n* = 52) had non-insulin-dependent diabetes mellitus, 3.5% (*n* = 49) had fatty liver disease, and 1.7% (*n* = 24) had other lipoprotein metabolism disorders.

The mean age was 37.31 years (standard deviation [SD] 10.29; 95% CI, 36.77–37.84 years; min. age 16 years and max. age 71 years), with a mean body mass index (BMI) of 40.3 kg/m² (SD 4.64; 95% CI 40.03–40.53 kg/m²; min. 25.00 – max. 67.90 kg/m²). Information about the BMI group to which the patient belonged at the time of surgery can be seen in Table 1.

**Table 1:** Disposition of patients according to the BMI group prior to bariatric surgery

BMI group	Mean BMI (kg/m²)	Cases % (*n*)
Overweight	27,4	0,53% (7)
Obesity Grade I	31,2	0,98% (13)
Obesity Grade II	37,0	48,44% (638)
Obesity Grade III	43,8	50,03% (659)
Overall Total	40,3	1317*

*Notes: There were patients who did not have this information, as it is not mandatory for procedure approval

Regarding the type of procedure involved in the surgery, preference for gastric bypass was observed in 88.9% (*n* = 1250) of cases compared to 11.1% (*n* = 156) for sleeve gastrectomy. The average length of stay for these patients was 1.84 days (SD 1.62; 95% CI 1.75 to 1.92 days; min. 0.2 – max. 35.3 days). However, the average length of stay for gastric bypass was 1.86 days, and for sleeve gastrectomy, it was 1.68 days. The results showed that the average length of stay did not differ significantly between the groups (*p* = 0.056) (Table 2).

**Table 2:** Difference in actual length of stay in surgical procedures

		Actual length of stay (days)			
		Mean	Standard deviation	Value – p*	Difference from averages [95% IC]
Surgical procedure	Sleeve	1,676	0,836	0,056	-0,1801 [-0,3207, -0,0150]
	Gastric bypass	1,856	1,688		

Note: * * Student’s t test for independent samples

Few acquired conditions during hospitalization were coded, with the most observed being vascular complications following infusion, transfusion, and therapeutic injection, 22.45% (*n* = 11), hemorrhage and hematoma complicating procedure not classified elsewhere, 8.16% (*n* = 4), leakage, 8.16% (*n* = 4). In addition, we did not observe any pulmonary embolism, or intestinal obstruction in our dataset. There was one death during this study period; however, it was not possible to establish any correlation with the performed surgery.

**Fig. 1:**
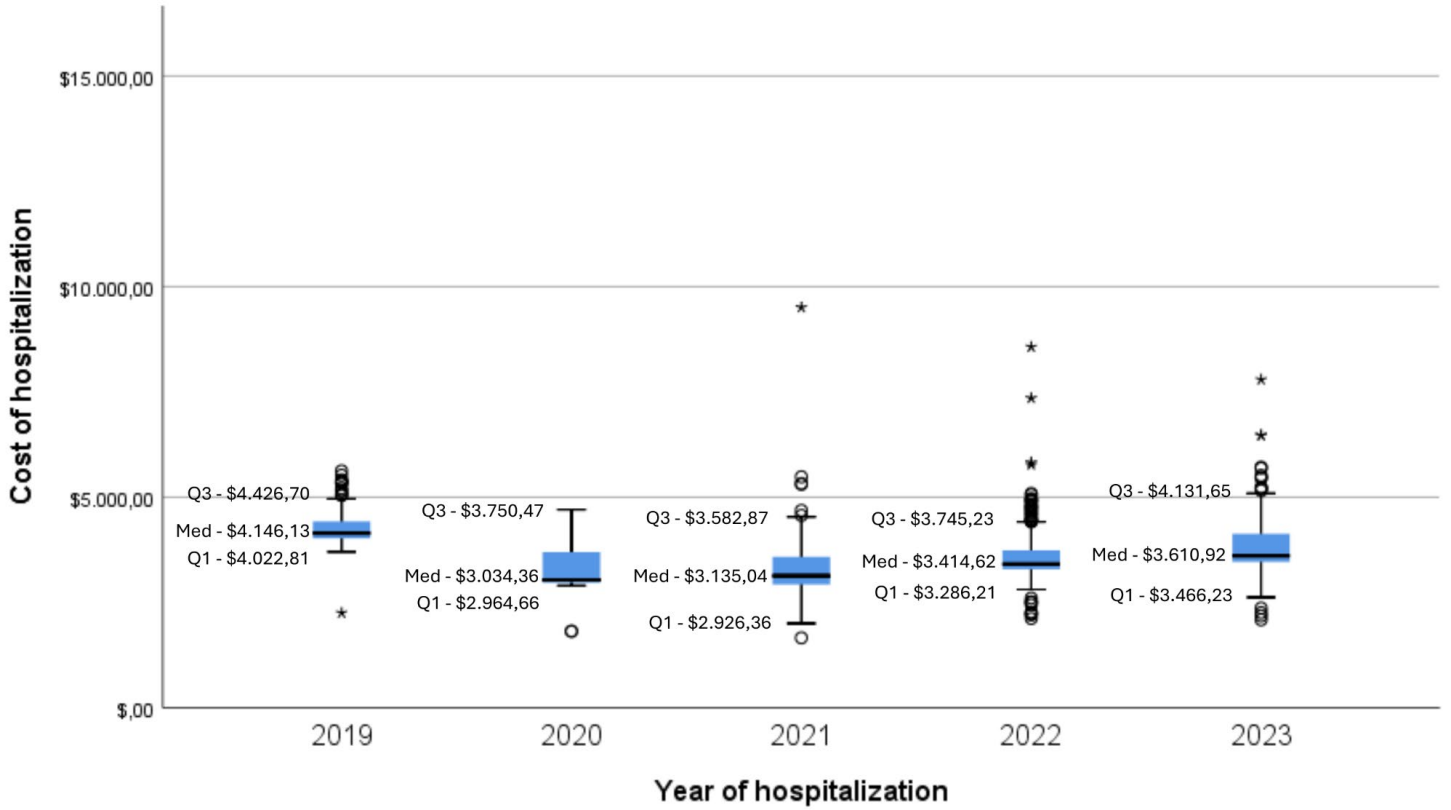
Evolution of the median cost during the hospitalization period from 2019 to July 2023, in the various healthcare providers coded by the health insurance company

The median of patients’ costs during hospitalization was $3,426.81 (Mean $ 3,615.19; SD $1,411.72; min. $1,660.82 – max. $37,081.21), and the trend of the median value over the years can be seen in Fig. 1. The main identified costs were materials and prostheses (32.7%), medical fees (27.4%), room rate (17.3%), daily charges (9.5%), and medications (7.3%).

The 30-day readmission rate was 7.7% in the obesity grade 1 BMI group (*n* = 1/14), 5.30% in the obesity grade 2 BMI group (*n* = 34/668), 4.9% in the obesity grade 3 BMI group (*n* = 32/693), and none in overweight patients. Differentiation among BMI groups may indicate the need for a more personalized approach in post-surgical monitoring, especially for patients with obesity grades 2 and 3. Despite the fact that 67 out of 72 readmitted patients (92.53%) had undergone bypass surgery, and 7.46% (*n* = 5) had sleeve surgery, Pearson’s chi-squared test did not reveal a significant association between BMI type and readmission rate (χ²(3) = 0.708, *p* = 0.871). Similarly, the likelihood ratio also did not indicate a significant association (χ²(3) = 1.040, *p* = 0.791).

The average costs of patients during the four years of the study up to one year after hospital discharge were also assessed. These patients were divided into two groups: those who were readmitted within 30 days and those who were not. The median annual cost during these four years for patients who were not readmitted was $353.51 (mean $702.03; SD $1,179.36; min. $0.00 – max. $16,219.92). For patients who had a readmission within 30 days, the median cost in the first year was $1,748.75 (mean $5,317.39; SD $10,658.66; min. $275.13 – max. $66,901.68).

**Fig. 2:**
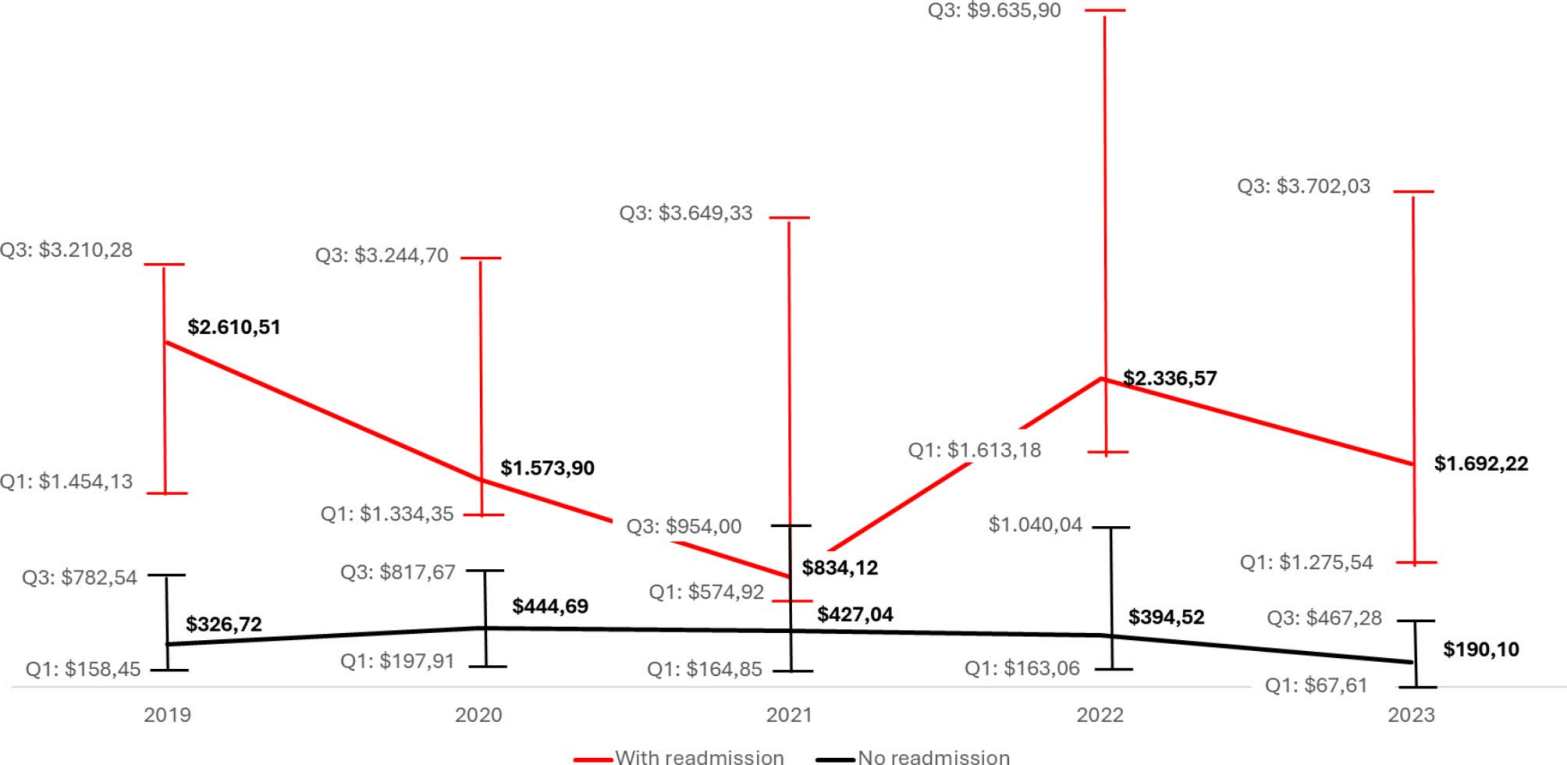
Evolution of the median cost of patients within one year after discharge from the hospital from 2019 to July 2023, in the different providers coded by the health operator

In other words, patients who were readmitted within 30 days had a median post-discharge cost 394.7% higher than those who were not readmitted. The four largest post-discharge costs for bariatric surgery patients are dominated by hospital stays, which account for 38.2% of the total expenses. Following this, general medications contribute 23.3%. Computed tomography scans represent the third highest cost at 3.8%, closely followed by room rate at 3.7%. Collectively, these components make up the majority of post-operative cost. The economic trend of median values over the years can be observed in Fig. 2.

**Fig. 3:**
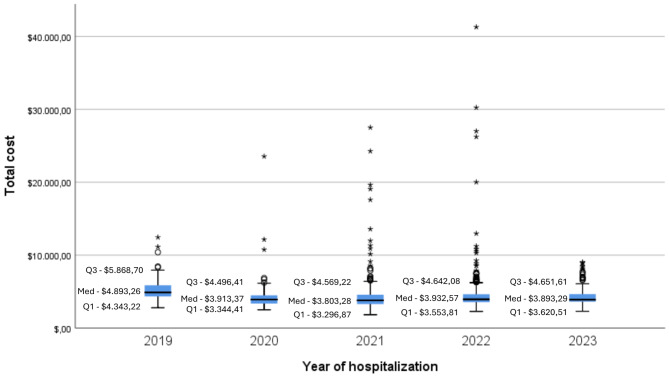
Evolution of the median total cost throughout the beneficiary’s post-procedure journ in the study period (2019 to 07/2023), in the different providers coded by the health operator

The total costs throughout the entire journey (hospitalization plus one-year post-procedure) of patients who underwent the procedure were also calculated. The median was $3.954,46 (mean $4,555.78; SD $3,196.13; min. $1,831.53 – max. $70,640.82). The economic trend of median values over the years can be observed in Fig. 3. An upward trend can be observed in the costs of 2019 compared to subsequent years, which can possibly be explained by two main factors: the higher number of days in 2019 and the appreciation of the dollar in 2020 and subsequent years, followed by a stabilization of values.

Furthermore, some correlations between the variables collected were tested, and Kendall’s correlation was used due to the lack of normal distribution of the data. The variables age and hospitalization cost showed a positive correlation (τ = 0.142), indicating that costs during hospitalization tend to increase with age. However, the shared variance between age and hospitalization cost is only 2.0%. The variable ‘DRG Complexity Brazil’ does not significantly correlate with any other variables (Table 3).

Post-discharge cost has a significant positive correlation with age (τ = 0.081**) and weight (τ = 0.103**), suggesting that costs after discharge tend to increase with age and weight. Age has a significant negative correlation with BMI (τ = -0.218**), suggesting that as age increases, BMI tends to decrease in study sample. All variables showed a weak correlation with each other (Table 3).

**Table 3:** Kendall’s tau correlation between the variables studied

Variable	BMI	Age	DRG Brazil Complexity	Cost of hospitalization	Post discharge cost	Weight
BMI						
Age	-0,218^**^					
DRG Brazil Complexity	-0,025	0,037				
Cost of hospitalization	-0,017	0,142^**^	0,060^*^			
Post discharge cost	-0,091^**^	0,081^**^	0,044	0,074^**^		
Weight	0,682^**^	-0,147^**^	-0,004	0,149^**^	0,103^**^	

Note: *The correlation is significant at the 0.05 level (2-tailed)

**Correlation is significant at the 0.01 level (2-tailed)

Moreover, we sought to investigate whether different BMI groups (BMI > = 40 versus BMI < 40) would influence the variables of length of stay, readmission, and cost. Assuming that variables had equal variances (Table S2), the t-test was conducted to assess the difference between these two suggested groups. However, no statistical difference was found between them in the evaluated variables (Table 4).

**Table 4:** T-Test bootstrapping to verify differences between BMI groups of > = 40 and < 40 for variables: length of stay, hospitalization cost, Post discharge cost and total cost

Variables	Groups	Mean	Standard Deviation	Value- *p*	Confidence interval (Lower and Upper)	
Length of stay (days)	BMI < 40	1,85	1,968	0,682	-0,1133	0,2199
	BMI > = 40	1,81	1,277			
Hospitalization Cost (UDS)	BMI < 40	3.759,70	1.448,47	0,601	-117,54	202,49
	BMI > = 40	3.718,45	1.515,96			
Post discharge cost (UDS)	BMI < 40	997,76	2.695,05	0,916	-330,70	358,20
	BMI > = 40	979,43	3.187,52			
Total cost (UDS)	BMI < 40	4.757,46	3.124,65	0,751	-307,90	457,99
	BMI > = 40	4,697.88	3,567.29			

Note: Bootstrapping results are based on 1000 samples

## Discussion

The search for effective approaches in treating obesity and its comorbidities has emphasized the growing importance of bariatric surgery [18]. In this context, the results extracted from DRGs presented in this research reveal valuable information regarding the performance of MBS in four major hospitals in the region during the investigated period.

A total of 281.6 bariatric surgeries were performed annually, indicating a considerable demand for this procedure in the studied region. The variation between the minimum of 87 and the maximum of 471 surgeries per year may suggest seasonal influences or the operational capacity of hospitals, considering that the number of coded hospitals varied during the study period. In 2021, the number of bariatric surgeries performed in Brazil was 63,016 procedures, with 57,152 through health insurance plans, 2,864 by Brazilian Unified Health System (SUS), and about 3,000 privately. In 2020, there were 52,715, with 46,437 bariatric surgeries through health insurance plans, 3,768 by SUS, and 2,510 privately [5].

The predominance of female patients, with an average of 74.8%, highlights a consistent trend over the years. This pattern may reflect not only the higher incidence of obesity in women, in line with national surveys, but also the fact that they more frequently seek surgical interventions for obesity treatment in this group [4, 5, 19].

The observed average age was 37.31 years, indicating that bariatric surgery is performed at a relatively young age. The age range, ranging from 16 to 71 years, highlights the diversity of patients seeking this type of intervention [4, 5, 19].

The BMI observed, varying from 25.00 to 67.90 kg/m2, covers a wide range of weight categories, indicating the inclusion of patients with different degrees of obesity, as also observed in Fink et al. (2022) [3]. This highlights the adaptability of bariatric surgery in managing a range of requirements, spanning from instances of excess weight to scenarios of morbid obesity. While patients in higher BMI groups may present additional challenges or distinct outcomes compared to those with less severe obesity [3, 19], from the outcomes analyzed in this study (acquired condition, procedure type, and rehospitalization), there was no statistical difference between the different BMI categories, even when highlighting the sample’s heterogeneity in terms of age and degree of obesity.

In a recent Brazilian study, during the months explored in the research, the overall proportion of female patients was 67.2% (915). Most patients were between 30 and 44 years old (688, 52.12%) at the time of the operation. The average age was 39 years (± 11.2 years). The baseline mean BMI was 41.5 kg/m² (± 6.9 kg/m²). Only 118 patients (8.9%) had a BMI over 50 kg/m². In four cases, the BMI was below 30, and in 98 cases, it was between 30.1 and 34.9 kg/m² [20].

The occurrence of non-insulin-dependent diabetes mellitus and fatty liver degeneration highlights the intrinsic relationship between obesity and other metabolic conditions. These results reinforce the importance of bariatric surgery not only in weight reduction but also in improving related metabolic conditions [2, 20–24]. In a meta-analysis, patients lost an average of 61% of excess weight after bariatric surgery; as a result, 77% of cases of diabetes mellitus were resolved, 62% of cases of hypertension were resolved, and 86% of cases of sleep apnea were resolved [25]. Studies emphasize that nutritional follow-up is crucial for the success of surgery, contributing to avoiding future complications and ensuring positive outcomes [24]. Therefore, continuous nutritional support plays an essential role in optimizing post-surgical outcomes.

The preference for gastric bypass over sleeve gastrectomy highlights a trend in the choice of surgical procedure in this study. Gastric bypass, also known as Roux-en-Y gastric bypass, involves creating a small pouch in the stomach, which is directly connected to the small intestine, bypassing part of the stomach and the initial intestine. It tends to result in more significant weight loss and a favorable impact on metabolic conditions, such as type 2 diabetes [3, 26, 27]. In sleeve gastrectomy, about 75% of the stomach is removed, leaving a tubular or “sleeve” shape. Unlike gastric bypass, there is no diversion of the gastrointestinal tract in this procedure. It also promotes substantial weight loss and improvements in metabolic conditions, albeit to a lesser extent compared to gastric bypass [3, 26, 27].

The choice between these procedures depends on various factors, including patient characteristics, preexisting medical conditions, and surgeon preferences [3, 26]. Each procedure has specific advantages and disadvantages, and the decision should be individualized to meet the patient’s needs and weight loss goals [27].

The average length of hospital stay of 1.84 (0.2 to 35.3) days indicates an efficient approach to postoperative management. The absence of statistical difference in the length of stay between gastric bypass and sleeve gastrectomy suggests that both techniques can be performed with similar efficiency, providing patients with a relatively quick recovery compared to some other surgical interventions [20, 28, 29]. In a study conducted in Australia with a sample of 63,604 bariatric procedures performed, the average length of hospital stay was 2.3 (standard deviation 1.31) days. The occurrence of any adverse event extended the stay by an additional 1.14 days [29].

The conditions acquired during hospitalization, although relatively few, included vascular complications, hemorrhages, hematomas and leakage. The observation of one death during the study period, although without an established correlation with the present study, highlights the complexity and risks associated with bariatric surgery. Regarding the 30-day rehospitalization rate, our study is consistent with current literature 3,8% (4.180 de 109.900 patients) in Ahmed et al. (2023) [30], 4,8% (18 of 378 patients) in Quadri et al. (2022) [31], 3,7% (18.860 of 509.631 patients) in Al-Mazrou et al. (2021) [32] e 7,5% (110 of 1468 patients) in Dang et al. (2020) [33].

The average total cost during hospitalization was $ 3,740.80; with a significant standard deviation of $ 1,472.42. This considerable variation highlights the heterogeneity in costs associated with bariatric surgery, reflecting the complexity of cases and potential complications. In the literature, few studies evaluate the profile of total healthcare costs for bariatric surgery patients. At the Central University Hospital of Asturias in Spain, the average cost per hospitalization calculated by DRG codes was 6,545.90€, and the average cost per patient was 10,572.20€ [8].

In the SUS, in 2017, the average cost during hospitalization was R$ 5,824.61 per bariatric surgery, with approximately 70% of this amount related to hospital services and 30% to professional services [28]. In the United States, hospital costs for bariatric surgery were higher (US$ 11,773.00) in the public healthcare system (Medicare and Medicaid) compared to private healthcare (US$ 4,435.00) [4]. In another study in the United States, evaluating 687,866 patients in 2,435 hospitals, the average annual cost of the procedure was US$ 10,900 (interquartile range: 8,600 − 14,000) for laparoscopic vertical sleeve gastrectomy and US$ 13,600 (10,300 − 18,000) for Roux-en-Y gastric bypass [34].

The costs of patients up to one year after hospital discharge were assessed, with a median cost of $ 26,974.73. The considerable range, from $ 1,484.29 to R$ 336,796.95, highlights the variability in postoperative costs, possibly related to complications, readmissions, and individual follow-up needs. In Chao et al. (2023) [35], the average direct costs after bariatric surgery for laparoscopic vertical sleeve gastrectomy in the first year were US$ 1,083.00 and for Roux-en-Y gastric bypass were US$ 1,228.00.

Several studies have examined how long it would take for surgery to pay off compared to medical costs attributable to obesity. Finkelstein and Brown (2005) [36] reported that it took 5 to 10 years to fully recover the costs associated with bariatric surgery. Meanwhile, Sampalis et al. (2004) [37] using Canadian medical cost data, reported that it took 3.5 years to break even when focusing solely on medical costs. In another study, Cremieux and Buchwald (2008) [38] stated that the average investment in bariatric surgery ranged from approximately US$ 17,000 to US$ 26,000 in the United States, and costs were estimated to be recovered in two years for laparoscopic surgery patients and in four years for open surgery patients. Two years is also cited in the study by Rodicio Miravalles et al. (2012) [8].

Wu et al. (2021) [23] comprehensively reviewed the evidence of cost-effectiveness of bariatric surgery, analyzing 28 complete economic studies. It was concluded that bariatric surgery is cost-effective in high-income countries, especially for patients with obesity and diabetes, over periods of 10 years and throughout life.

In another recent study estimating the total cost of bariatric surgery in Denmark and its impact on healthcare costs and labor market insertion up to five years after surgery, using national registry data, patients who underwent bariatric surgery were compared with matched controls [39]. Despite the Danish healthcare system, and consequently, the payer perspective being different from that of Brazil, we aim to conduct similar evaluations in the perspective of the Brazilian supplementary healthcare system in the future.

There are some limitations in this work as it is a retrospective survey of healthcare and direct costs generated by events in a health insurer, such as: Market factors related to the coverage and costs of bariatric surgery procedures will affect the results, and the costs raised are related to the payer’s event database; Patient selection bias, the study only captured beneficiaries undergoing bariatric surgery by DRG-coded providers, which may limit the generalization of the results; The study’s results may not be generalizable to other populations or healthcare systems, especially as they were based on data from a single health insurer; Lack of control for confounding variables, there may be uncontrolled variables influencing the results, such as additional patient comorbidities, adherence to postoperative treatment, and lifestyle changes, which are not fully addressed in the study; Limitations in assessing quality of care: Although our study measures improvement in quality of care by acquired conditions and readmission, there are other variables that could be considered, but these are not yet quantifiable for evaluating the quality of care provided to patients undergoing bariatric surgery.

## Conclusion

In summary, the study found that female patients are more common among those undergoing bariatric surgery in the four major hospitals of the region. Most patients had grade II or III obesity, with an average age of 37.3 years and an average BMI of 40.3 kg/m². Gastric bypass was the most frequently performed procedure, making up 88.9% of cases. The length of hospital stay was similar across different procedures. Common complications included vascular issues, bleeding and leakage. The readmission rate within 30 days varied with different BMI levels. Overall costs, including hospitalization and follow-up for a year, were high and increased with patient age. The relationships between different factors like age, weight, and costs were generally weak, suggesting other influencing factors. The results underscore the complexity of bariatric surgery and the need for tailored postoperative care and resource management to improve both clinical and economic outcomes.

## Electronic supplementary material

Below is the link to the electronic supplementary material.

Supplementary Material 1

## Data Availability

The datasets used and/or analysed during the current study are available from the corresponding author on reasonable request.
