# Supplementary material for: Evaluation of the cost and care outcomes by group related to the diagnosis of bariatric surgery
Source: BMC Surg. 2024 Nov 29;24:381. doi: 10.1186/s12893-024-02682-y (PMC11606067; doi:10.1186/s12893-024-02682-y)
Supplement: Supplementary file 1 — Supplementary Material 1 [file 12893_2024_2682_MOESM1_ESM.docx]

SUPPLEMENTAL MATERIAL

Table S1. Number of patients by sex who underwent bariatric surgery during the period from 2019 to 2023.

| Year | Female (n) | Male (n) | NI* | General Total |
| --- | --- | --- | --- | --- |
| 2019 | 71,3% (62) | 28,7% (25) |  | 87 |
| 2020 | 71,4% (70) | 28,6% (28) |  | 98 |
| 2021 | 76,3% (312) | 23,5% (96) | 1 | 409 |
| 2022 | 75,8% (357) | 24,20% (114) |  | 471 |
| 2023 | 73,5% (252) | 26,53% (91) |  | 343 |
| General Total | 74,8% (10530) | 25,14% (354) | 1 | 1408 |

NI. Not identified

Table S2. Levene's test to verify the equality of variances in the variables: Actual length of stay, readmission, and total cost.

|  | Levene's test for equality of variances | |
| --- | --- | --- |
|  | Z | Value - p |
| Hospitalization costs | 0,044 | 0,834 |
| Post discharge cost | 0,031 | 0,861 |
| Total cost | 0,054 | 0,816 |
| Length of stay | 1,841 | 0,175 |
